# A Potential Autophagy-Related Competing Endogenous RNA Network and Corresponding Diagnostic Efficacy in Schizophrenia

**DOI:** 10.3389/fpsyt.2021.628361

**Published:** 2021-02-23

**Authors:** Rongjie Li, Qiaoye Wang, Yufen Qiu, Youshi Meng, Lei Wei, Hao Wang, Ruikang Mo, Donghua Zou, Chunbin Liu

**Affiliations:** ^1^Department of Neurology, The Fifth Affiliated Hospital of Guangxi Medical University, Nanning, China; ^2^Department of Internal Medicine, The Fifth Affiliated Hospital of Guangxi Medical University, Nanning, China; ^3^Maternal and Child Health Hospital and Obstetrics and Gynecology Hospital of Guangxi Zhuang Autonomous Region, Guangxi, China

**Keywords:** schizophrenia, autophagy, ceRNA, competing endogenous RNA, lncRNA-miRNA-mRNA

## Abstract

Competing endogenous RNA (ceRNA) and autophagy were related to neurological diseases. But the relationship among ceRNA, autophagy and Schizophrenia (SZ) was not clear. In this study, we obtained gene expression profile of SZ patients (GSE38484, GSE54578, and GSE16930) from Gene Expression Omnibus (GEO) database. Then we screened the autophagy-related differentially expressed lncRNA, miRNA, and mRNA (DElncRNA, DEmiRNA, and DEmRNA) combined with Gene database from The National Center for Biotechnology Information (NCBI). In addition, we performed enrichment analysis. The result showed that biological processes (BPs) mainly were associated with cellular responses to oxygen concentration. The enriched pathways mainly included ErbB, AMPK, mTOR signaling pathway and cell cycle. Furthermore, we constructed autophagy-related ceRNA network based on the TargetScan database. Moreover, we explored the diagnostic efficiency of lncRNA, miRNA and mRNA in ceRNA, through gene set variation analysis (GSVA). The result showed that the diagnostic efficiency was robust, especially miRNA (AUC = 0.884). The miRNA included hsa-miR-423-5p, hsa-miR-4532, hsa-miR-593-3p, hsa-miR-618, hsa-miR-4723-3p, hsa-miR-4640-3p, hsa-miR-296-5p, and hsa-miR-3943. The result of this study may be helpful for deepening the pathophysiology of SZ. In addition, our finding may provide a guideline for the clinical diagnosis of SZ.

## Introduction

Schizophrenia is a serious genetic psychiatric disease that usually occurs in late adolescence or early adulthood, and it affected 1.13 million people worldwide in 2017 (1, 2). Lifetime prevalence of the disease is close to 1%, and only 10–15% of patients are able to engage in paid work (3). The main risk factors of the disease include disorders of the dopamine system (4); early brain trauma, especially damage to the frontal and temporal lobes (5); use of illicit drugs (6); and infections during pregnancy caused by various factors (7). The pathogenesis of schizophrenia is unclear, and most studies have shown it to involve interactions between genes and the environment (8). The disease is diagnosed based on positive symptoms, such as hallucinations, delusions, and unusual behavior; or negative symptoms, such as blunted emotional reactions, lack of emotion and lack of language. The presence of two or more symptoms is usually indicative of the disease. First-line treatments against schizophrenia are haloperidol and chlordiazepoxide, but they often show poor efficacy and are associated with high risk of serious adverse reactions (9). Identifying better treatments requires a deeper understanding of the biological basis of schizophrenia.

Autophagy, the process of degrading intracellular components in lysosomes, plays an important role in the central nervous system by contributing to neuronal homeostasis (10). Loss of autophagy can destroy neuronal homeostasis (11), leading to abnormal neuronal activity, which in turn may contribute to various neurological disease (12). In fact, loss of autophagy in animal model can seriously damage social and cognitive functions, which may lead to mood disorders, psychotic-like symptoms and behavioral change (13, 14). Dysregulation of autophagy in neurological diseases may involve altered gene regulation. In particular, it may involve changes in how much microRNAs (miRNAs) repress the translation of target genes, perhaps as a result of changes in the levels of long non-coding RNAs (lncRNAs) (15). According to the competing endogenous RNA (ceRNA) hypothesis, lncRNAs compete with target mRNAs for binding to miRNAs, acting as miRNA “sponges” (16). In support of this hypothesis, altered lncRNA-mediated gene regulation has been implicated in schizophrenia (17), and certain miRNAs are up-regulated in schizophrenia and other neurological diseases (18).

Whether schizophrenia involves altered interactions among lncRNAs, miRNAs, and mRNAs is unclear. Based on comparison of blood samples from schizophrenia patients and healthy controls in public datasets, the present study identified a ceRNA network that may regulate autophagy-related genes in the disease. These insights may help clarify the disease process, guide new drug development, and improve diagnosis.

## Materials and Methods

### Data Collection and Processing

**Figure 1:**
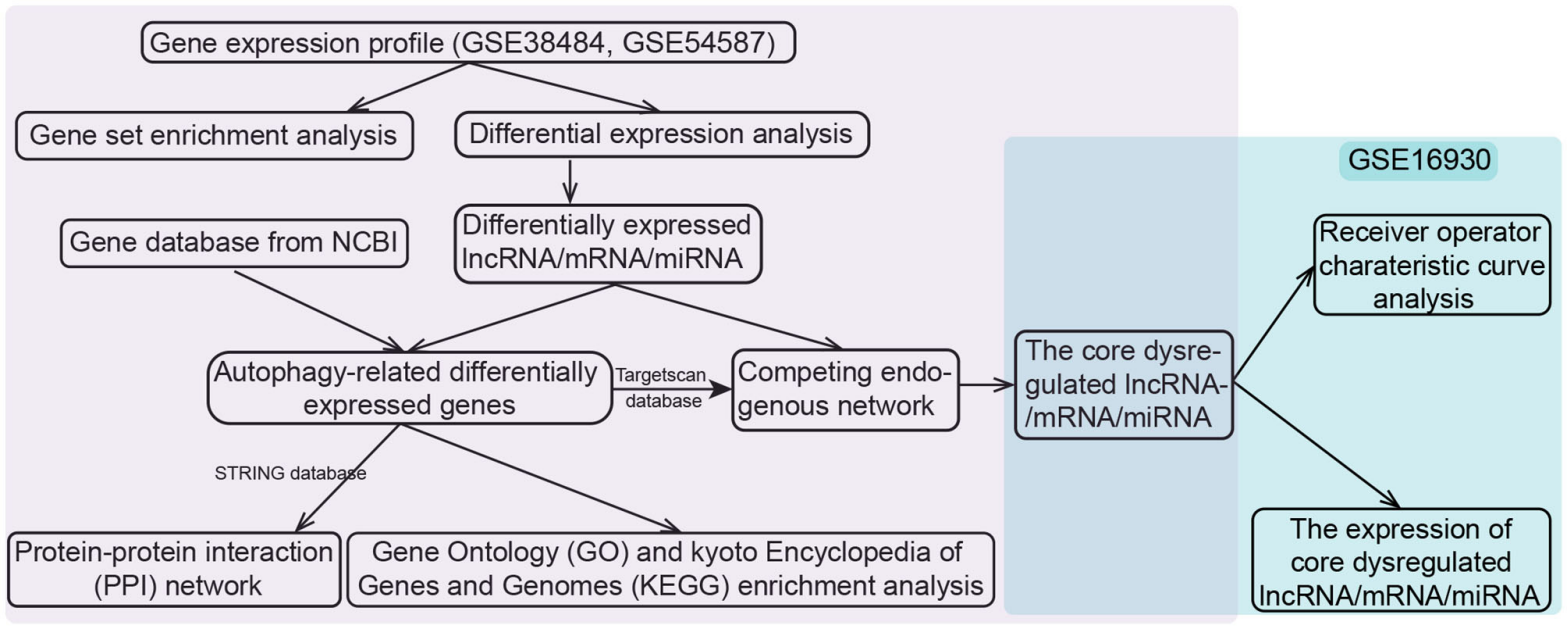
Flowchart of the study design. lncRNA, long noncoding RNA; miRNA, microRNA; NCBI, National Center for Biotechnology Information.

We downloaded the datasets from the Gene Expression Omnibus (GEO) database, each dataset had been normalized with MAS5 when the authors submitted them into the database as required (http://www.ncbi.nlm.nih.gov/geo/). The whole-blood RNA (mRNAs and lncRNAs) expression profiles of GSE38484 based on GPL6947 platform, taken from 106 patients with schizophrenia and 96 controls (19, 20). Peripheral-blood miRNA expression profiles of GSE54578 based on GPL16016 platform included 15 patients and 15 controls were also downloaded (21). The lncRNAs and mRNAs were distinguished according to the file *Homo_sapiens.GRCh38.97.chr.gtf* on the Ensembl website (http://asia.ensembl.org) (22, 23). The above two datasets (GSE38484 and GSE54587) were used to construct a potential ceRNA network in schizophrenia. The dataset of GSE16930 based on GPL2879 platform, containing 18 patients and 2 controls (24), was used to validate diagnostic performance and expression of RNA in ceRNA network. If one gene corresponded to multiple probes, the average expression value the these probes was considered to be the expression of the gene. The work flow was shown in Figure 1.

### Screening for Autophagy-Related Differentially Expressed RNAs in Schizophrenia

The *limma* package (25) was used to identify differentially expressed mRNAs, lncRNAs, and miRNAs (DEmRNA, DElncRNA, and DEmiRNA) between patients with schizophrenia and controls. The RNA that was log_2_|fold change (FC)| >1 and adjusted *p* < 0.05 was considered differentially expressed. The autophagy-related genes were obtained combined the differentially expressed RNAs (DERNAs) and the autophagy-related genes in Gene database (www.ncbi.nlm.nih.gov/gene). The autophagy-related genes were obtained using the autophagy as the search key word in the Gene database.

### Functional Enrichment Analysis

Potential interactions among autophagy-related genes were identified using the STRING database (26), and protein-protein interactions (PPIs) network was visualized using Cytoscape (27). In order to further explore the biological functions of autophagy-related genes, gene ontology (GO) and the Kyoto Encyclopedia of Genes and Genomes (KEGG) enrichment analysis was performed using the c*lusterProfiler R* package (28). For further exploring the differences of biological functions between SZ patients and controls, the gene set variation analysis (GSVA) was performed using GSVA package (29) in R. Gene set enrichment analysis (GSEA) was performed using GSEA2-2.2.4 (Java version) (30). The reference gene set (*c5.bp.v6.2.symbols.gmt* and *c2.cp.kegg.v6.2.symbols.gmt*) were obtained from The Molecular Signatures Database (version 6.2) (31). GO and KEGG networks were analyzed and drawn using the *ClueGO* plug-in (32) in Cytoscape.

### Exploration of an Autophagy-Related ceRNA Network in Schizophrenia

To construct a ceRNA regulation network, interactions between DEmRNAs and DEmiRNAs were predicted using the TargetScan database (version: release 7.2) (33). Then the DEmiRNAs in these interaction pairs were used to identify target mRNAs, again based on the TargetScan database. Target mRNAs that we found to be differentially expressed in schizophrenia were considered candidate target mRNAs in the ceRNA network. The co-expression network comprising DElncRNAs, DEmiRNAs and DEmRNAs was visualized using Cytoscape.

### Identifying Core Dysregulated DElncRNAs, DEmiRNAs, and DEmRNAs in Schizophrenia

GSVA scores were calculated using an unsupervised, non-parametric algorithm in the GSVA package (29) separately for DElncRNAs, DEmiRNAs, and DEmRNAs. Core genes are also called hub genes, genes that play a vital role in biological processes. In related pathways, the regulation of other genes is often affected by this gene. The ability of the core sets identified based on GSVA score to diagnose schizophrenia was assessed in terms of the area under the receiver operator characteristic curve (AUC) (34).

### Statistical Analysis

We screened the differentially expressed genes in the two groups using unpaired *t*-tests provided by limma package. Unless otherwise stated, we considered *p*-value < 0.05 to be statistically significant.

## Results

### DElncRNAs, DEmiRNAs, and DEmRNAs in Schizophrenia

**Figure 2:**
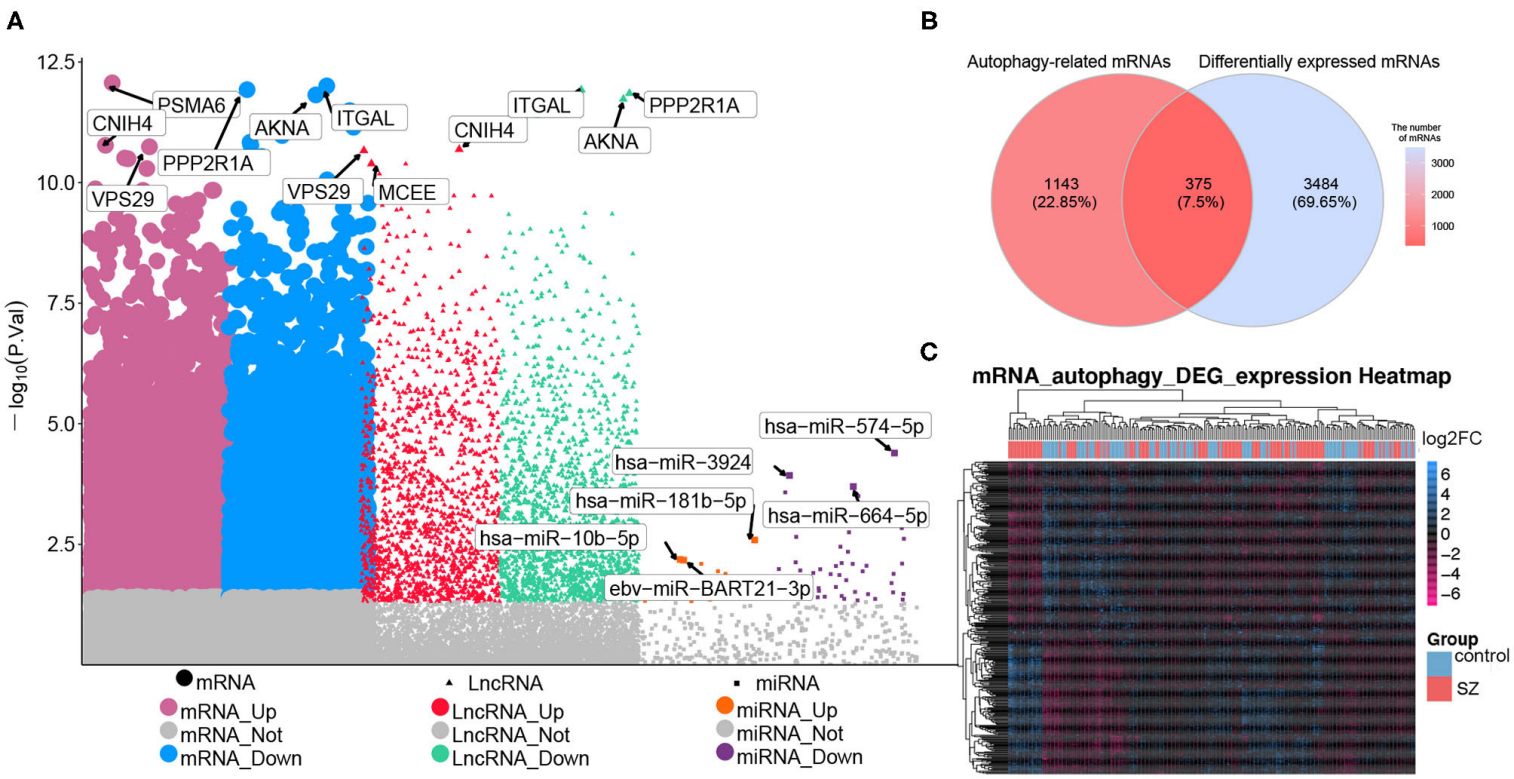
Differential expression analysis and screening of autophagy-related mRNAs. **(A)** Manhattan diagram showing differentially expressed (DE) lncRNAs, DEmiRNAs and DEmRNAs in schizophrenia (SZ). **(B)** Genes overlapping between the set of autophagy genes and the set of DEmRNAs. **(C)** Heatmap showing the expression of autophagy-related DEmRNAs. Yellow means up-regulation, blue means down-regulation.

Comparison between patients with schizophrenia and controls revealed 2,400 DElncRNAs (1,130 up-regulated, 1,270 down-regulated), 69 DEmiRNAs (19 up-regulated, 50 down-regulated), and 3,859 DEmRNAs (811 up-regulated, 2,048 down-regulated) (Figure 2A). Of the total set of DEmRNAs, 375 were related to autophagy, of which 176 were up-regulated and 199 down-regulated (Figure 2B). The heatmap suggested that DEmRNAs could distinguish patients from controls to a certain extent (Figure 2C).

### Biological Functions and Pathways Involving Autophagy-Related DEmRNAs in Schizophrenia

**Figure 3:**
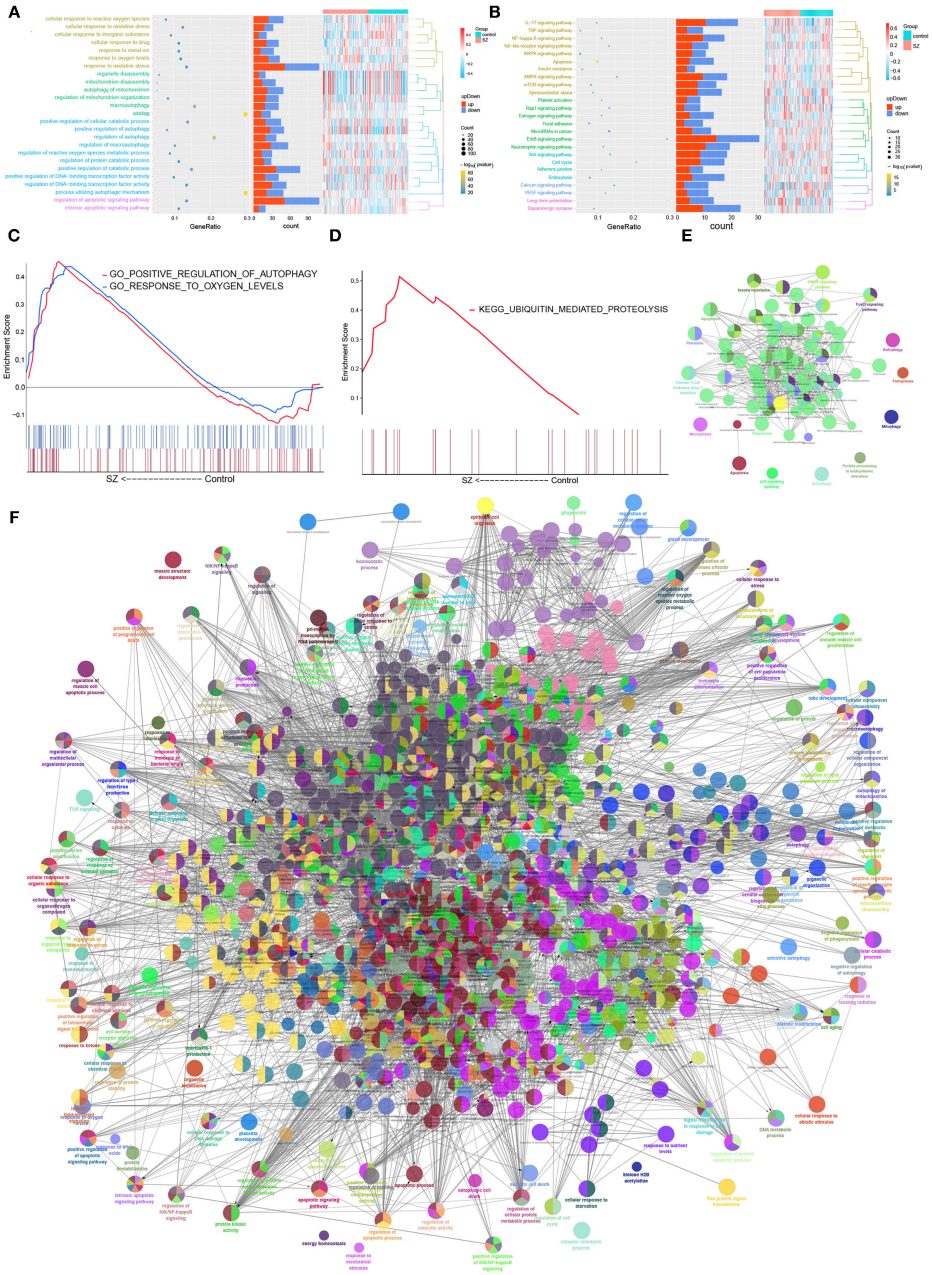
Biological functions and KEGG pathways involving autophagy-related DEmRNAs in schizophrenia (SZ). **(A)** Biological processes involving autophagy-related DEmRNAs. **(B)** Kyoto Encyclopedia of Genes and Genomes (KEGG) pathways involved in autophagy-related DEmRNAs. **(C)** Biological processes involving autophagy-related DEmRNAs. **(D)** KEGG pathways involved in autophagy-related DEmRNAs. **(E)** Pathway enrichment analysis of 375 autophagy-related DEmRNAs using ClueGO. **(F)** Biological enrichment analysis of 375 autophagy-related DEmRNAs using ClueGO. Each node represents a biological process or KEGG pathway. Edges represent connections between the nodes, and the length of each edge reflects the relatedness of two processes.

The autophagy-related DEmRNAs encoded a wide range of proteins, based on the STRING database. The analysis identified 161 interaction pairs and 130 nodes in the network when the score was higher than 980 (Supplementary Figure 1). Enrichment analysis showed that autophagy-related DEmRNAs supported cellular responses to oxidative stress, regulation of protein catabolism, apoptosis signaling, as well as biological processes related to cellular responses to oxygen concentration (Figure 3A). They were also closely related to ErbB signaling, AMPK signaling, mTOR signaling and the cell cycle (Figure 3B). The GSEA result showed that there were common GO function and KEGG pathways combined with Figures 3B,C. Two GO functions, “positive regulation of autophagy” and “response to oxygen levels,” were up-regulated in SZ patients compared with controls (Figure 3C). Only one KEGG pathway, “ubiquitin mediated proteolysis,” was upregulated in SZ patients compared to controls (Figure 3D). ClueGO analysis showed that autophagy-related DEmRNAs may also be related to apoptosis and to signaling mediated by mTOR, MAPK, and ErbB (Figure 3E). The results showed that autophagy-related DEmRNAs may be involved in positive regulation of catabolism, apoptosis signal, and regulation of transcription factors (Figure 3F).

### Involvement of a ceRNA Network in Autophagy-Related DEmRNAs in Schizophrenia

**Figure 4:**
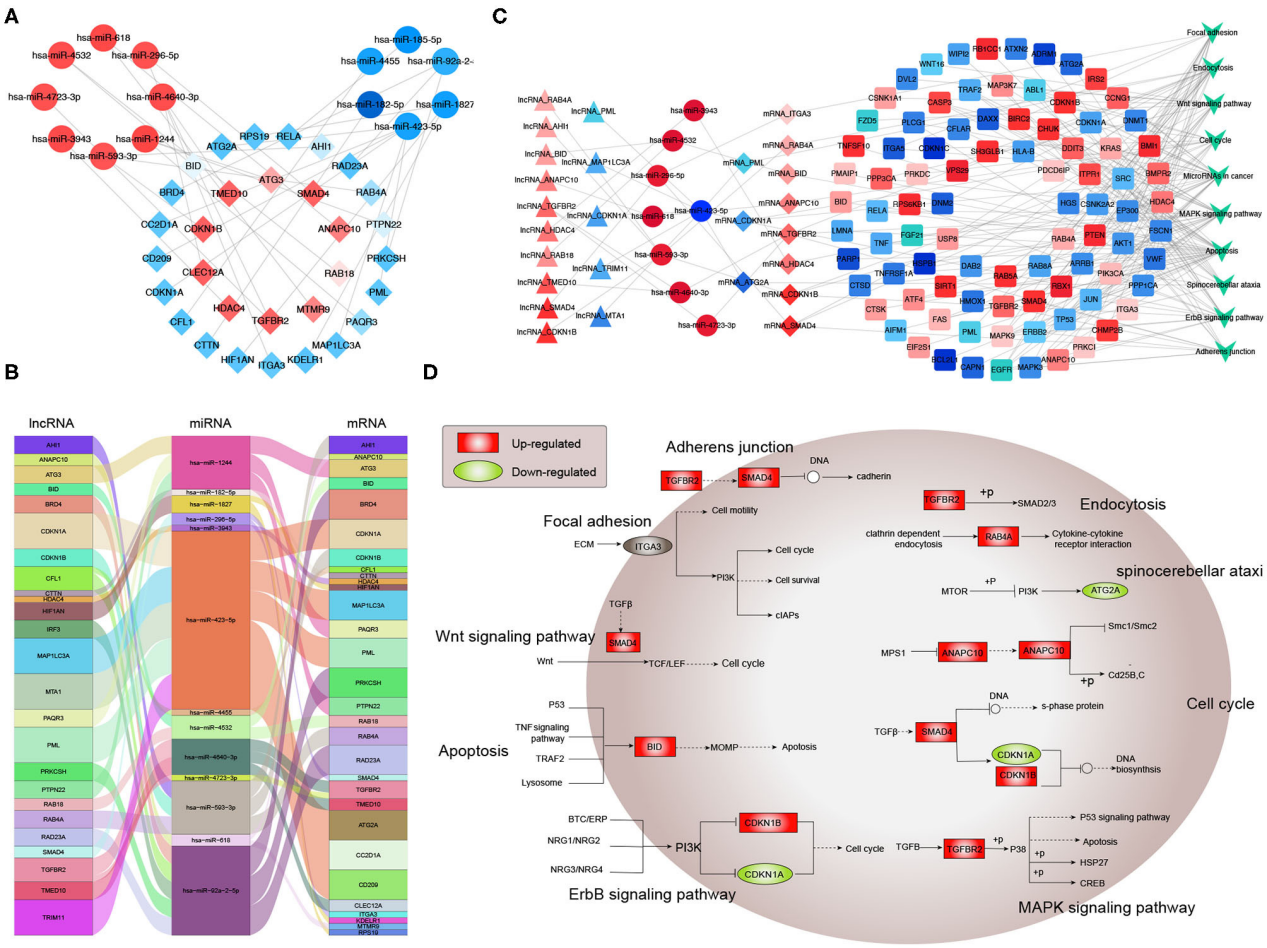
Exploration of a competing endogenous (ce) RNA network in schizophrenia. **(A)** Network of miRNAs, showing relationships between 14 miRNAs and 31 target genes (mRNAs). Red means up-regulation and blue means down-regulation. Circles indicate miRNAs; diamonds, mRNAs. The darker the color, the greater the absolute value of logFC. **(B)** Sankey plot, showing relationships among mRNAs, lncRNAs, and miRNAs. **(C)** Network of lncRNAs, miRNAs, mRNAs, and KEGG pathways. Relationships involving 8 miRNAs, 15 lncRNAs, 11 mRNAs, and 10 KEGG pathways are shown. Triangles represent lncRNAs; circles, miRNAs; diamonds, mRNAs; squares, genes in KEGG pathways; V,KEGG pathway. Blue means down-regulation; red means up-regulation. **(D)** Nine KEGG pathways in the ceRNA network that have previously been associated with schizophrenia.

Next, potential interactions among the above genes were explored according to the ceRNA hypothesis. Based on a minimal score of 0.4, we identified 31 autophagy-related DEmRNAs that may interact with 14 DEmiRNAs (Figure 4A, Tables 1–3). In total, there were 25 DElncRNAs, 13 DEmiRNAs and 30 autophagy-related DEmRNAs, with the threshold of score >0.4 (Figure 4B, Table 4). These results, combined with the enrichment analysis, suggest that lncRNAs may regulate the phenotype through ceRNA. We identified 15 DElncRNAs, 8 DEmiRNAs and 11 autophagy-related DEmRNAs and 10 KEGG pathways (Figure 4C, Tables 4, 5). We focused on the nine KEGG pathways previously linked to schizophrenia in the literature: Wnt signaling pathway, adherence junctions, ErbB signaling pathway, spinocerebellar ataxia, apoptosis, MAPK signaling pathway, cell cycle, endocytosis, and focal adhesion (Figure 4D).

**Table 1:** The differentially expressed miRNA in miRNA-mRNA interaction.

**Symbol**	**UP-/Down-regulated**	**logFC**
hsa-miR-4723-3p	Up	0.59274912
hsa-miR-185-5p	Down	−1.3649045
hsa-miR-296-5p	Up	0.57575869
hsa-miR-1827	Down	−1.1462577
hsa-miR-3943	Up	0.73822174
hsa-miR-182-5p	Down	−1.6125898
hsa-miR-4640-3p	Up	0.71116123
hsa-miR-4455	Down	−1.0447678
hsa-miR-92a-2-5p	Down	−0.6899372
hsa-miR-4532	Up	0.72144573
hsa-miR-1244	Up	0.84447821
hsa-miR-423-5p	Down	−1.0443911
hsa-miR-618	Up	0.80561009
hsa-miR-593-3p	Up	0.54746291

*MiRNA, microRNA; FC, fold change*.

**Table 2:** The differentially expressed mRNA in miRNA-mRNA interaction.

**Symbol**	**UP/Down-regulated**	**LogFC**
TMED10	UP	0.146429157
TGFBR2	UP	0.127977524
SMAD4	UP	0.18994502
RPS19	Down	−0.114099999
RELA	Down	−0.059082554
RAD23A	Down	−0.201071758
RAB4A	UP	0.065208968
RAB18	UP	0.087381128
PTPN22	UP	0.074606658
PRKCSH	Down	−0.131266843
PML	Down	−0.026899247
PAQR3	UP	0.062766367
MTMR9	UP	0.104245647
MAP1LC3A	Down	−0.074642569
KDELR1	Down	−0.111501156
ITGA3	UP	0.034938843
HIF1AN	Down	−0.103443561
HDAC4	UP	0.115260925
CTTN	Down	−0.080633062
CLEC12A	UP	0.219451641
CFL1	Down	−0.139858864
CDKN1B	UP	0.183884617
CDKN1A	Down	−0.09060451
CD209	Down	−0.026337891
CC2D1A	Down	−0.041592996
BRD4	Down	−0.03672642
BID	UP	0.077208773
ATG3	UP	0.097968842
ATG2A	Down	−0.166212351
ANAPC10	UP	0.10559715
AHI1	UP	0.071109368

*FC, fold change*.

**Table 3:** The cumulative weighted context++ score for miRNA targeted DEmRNAs.

**DElncRNA**	**DEmiRNA**	**Score**
PAQR3	hsa-miR-1244	0.432
ATG3	hsa-miR-1244	0.42
PTPN22	hsa-miR-1244	0.487
SMAD4	hsa-miR-296-5p	0.403
HDAC4	hsa-miR-3943	0.475
BID	hsa-miR-4532	0.437
RAB18	hsa-miR-4532	0.423
TGFBR2	hsa-miR-4640-3p	0.511
TMED10	hsa-miR-4640-3p	0.65
TGFBR2	hsa-miR-4723-3p	0.453
CDKN1B	hsa-miR-593-3p	0.484
AHI1	hsa-miR-593-3p	0.401
RAB4A	hsa-miR-593-3p	0.555
ANAPC10	hsa-miR-618	0.461
CTTN	hsa-miR-182-5p	0.842
HIF1AN	hsa-miR-1827	0.797
MTA1	hsa-miR-423-5p	0.646
PML	hsa-miR-423-5p	0.755
MAP1LC3A	hsa-miR-423-5p	0.974
TRIM11	hsa-miR-423-5p	0.635
CDKN1A	hsa-miR-423-5p	0.826
CFL1	hsa-miR-4455	0.928
RAD23A	hsa-miR-92a-2-5p	1.032
IRF3	hsa-miR-92a-2-5p	0.681
BRD4	hsa-miR-92a-2-5p	0.836
CFL1	hsa-miR-92a-2-5p	0.615
PRKCSH	hsa-miR-92a-2-5p	0.7

*DE, differentially expressed*.

**Table 4:** The cumulative weighted context++ score for miRNA targeted DElncRNAs.

**DEmiRNA**	**DEmRNA**	**Score**
hsa-miR-1244	ATG3	0.42
hsa-miR-1244	PAQR3	0.432
hsa-miR-1244	PTPN22	0.487
hsa-miR-296-5p	SMAD4	0.403
hsa-miR-296-5p	ITGA3	0.458
hsa-miR-3943	HDAC4	0.475
hsa-miR-4532	RAB18	0.423
hsa-miR-4532	BID	0.437
hsa-miR-4640-3p	TGFBR2	0.511
hsa-miR-4640-3p	CLEC12A	0.425
hsa-miR-4640-3p	TMED10	0.65
hsa-miR-4723-3p	TGFBR2	0.453
hsa-miR-593-3p	AHI1	0.401
hsa-miR-593-3p	CDKN1B	0.484
hsa-miR-593-3p	RAB4A	0.555
hsa-miR-618	MTMR9	0.452
hsa-miR-618	ANAPC10	0.461
hsa-miR-423-5p	ATG2A	1.259
hsa-miR-92a-2-5p	RAD23A	1.032
hsa-miR-423-5p	MAP1LC3A	0.974
hsa-miR-4455	CFL1	0.928
hsa-miR-185-5p	RELA	0.846
hsa-miR-182-5p	CTTN	0.842
hsa-miR-92a-2-5p	BRD4	0.836
hsa-miR-423-5p	CDKN1A	0.826
hsa-miR-1827	KDELR1	0.806
hsa-miR-1827	HIF1AN	0.797
hsa-miR-423-5p	PML	0.775
hsa-miR-423-5p	CC2D1A	0.71
hsa-miR-92a-2-5p	PRKCSH	0.7
hsa-miR-1827	RPS19	0.697
hsa-miR-423-5p	CD209	0.695

*DE, differentially expressed*.

**Table 5:** The differentially expressed lncRNA in lncRNA-miRNA-mRNA interaction.

**Symbol**	**UP-/Down-regulated**	**LogFC**
PAQR3	Up	0.06276637
ATG3	Up	0.09796884
PTPN22	Up	0.07460666
SMAD4	Up	0.18994502
HDAC4	Up	0.11526093
BID	Up	0.07720877
RAB18	Up	0.08738113
TGFBR2	Up	0.12797752
TMED10	Up	0.14642916
CDKN1B	Up	0.18388462
AHI1	Up	0.07110937
RAB4A	Up	0.06520897
ANAPC10	Up	0.10559715
CTTN	Down	−0.0806331
HIF1AN	Down	−0.1034436
MTA1	Down	−0.1248468
PML	Down	−0.0268992
MAP1LC3A	Down	−0.0746426
TRIM11	Down	−0.0777342
CDKN1A	Down	−0.0906045
CFL1	Down	−0.1398589
RAD23A	Down	−0.2010718
IRF3	Down	−0.2632613
BRD4	Down	−0.0367264
PRKCSH	Down	−0.1312668

*lncRNA, Long non-coding RNA; FC, fold change*.

### Diagnostic Ability of Autophagy-Related Core DElncRNAs, DEmiRNAs and DEmRNAs for Schizophrenia

**Figure 5:**
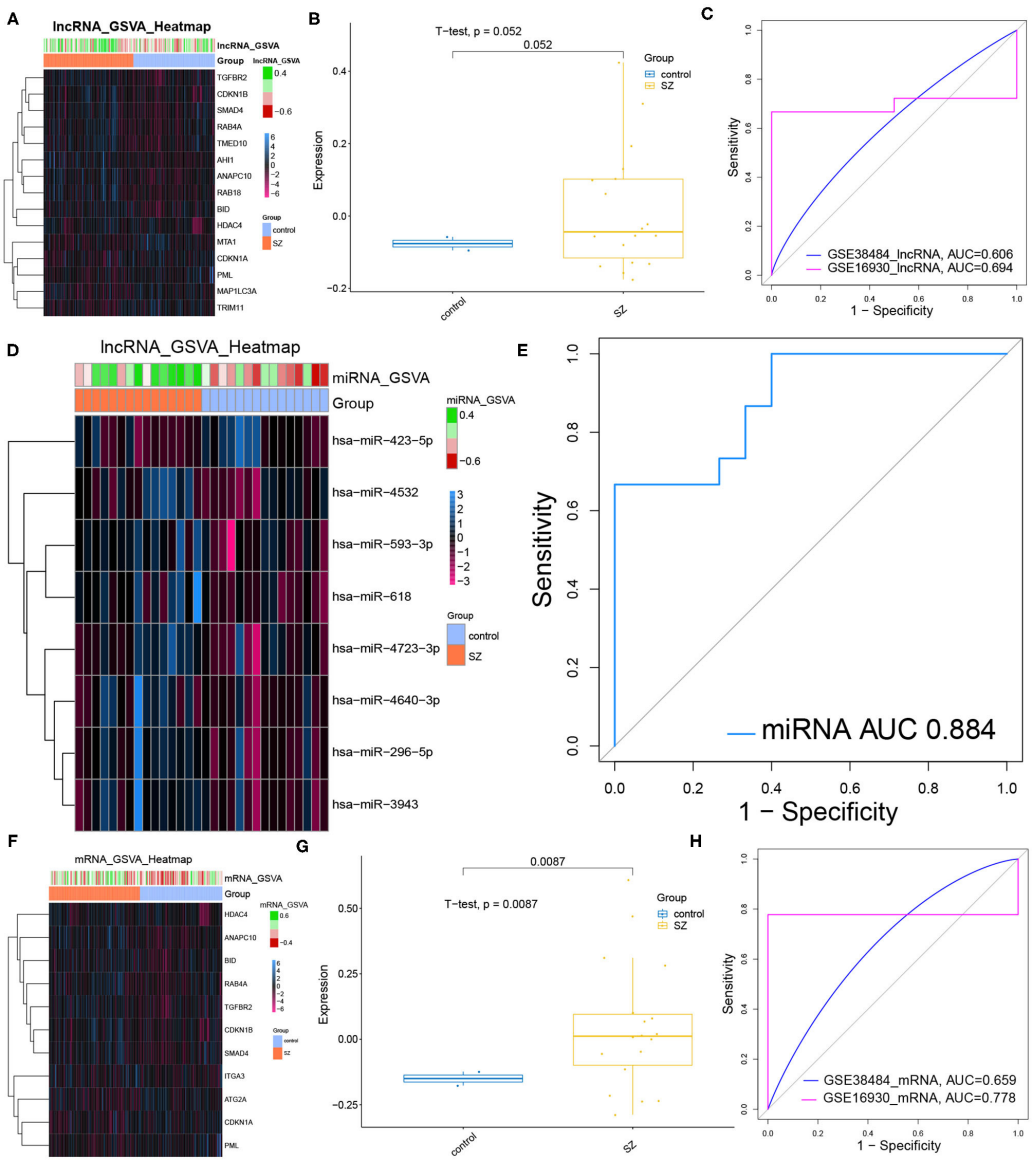
Performance of core dysregulated autophagy-related DElncRNAs, DEmiRNAs and DEmRNAs for diagnosing schizophrenia (SZ). **(A)** Gene set variation analysis (GSVA) of lncRNA expression. Blue means up-regulation; red means down-regulation. **(B)** GVSA score for core dysregulated DElncRNAs in the validation set (GSE16930). The horizontal axis shows sample names; the vertical axis, gene expression. Control data are shown in blue, patient data in yellow. **(C)** Receiver operating characteristic curves assessing how well the GSVA score for core dysregulated DElncRNAs diagnosed schizophrenia in the test set (GSE38484) and validation set (GSE16930). **(D)** GSVA-miRNA expression heat map. **(E)** ROC curve analysis for the GSVA score of core dysregulated DEmiRNAs in test set (GSE38484). **(F)** GSVA-mRNA expression heat map. **(G)** The expression of the GVSA score of core dysregulated mRNAs in validation set (GSE16930). **(H)** ROC analysis for the GSVA score of core dysregulated DEmRNAs in test set (GSE38484) and validation set (GSE16930).

Most core dysregulated DElncRNAs were up-regulated in schizophrenia compared to controls (Figure 5A). However, the GSVA score based on the core DElncRNAs did not differ significantly between patients and controls (Figure 5B, Table 5). Similarly, the core DElncRNAs showed a poor ability to differentiate patients from controls in the test set (GSE38484, AUC = 0.606) and validation set (GSE16930, AUC = 0.694) (Figure 5C). Although core dysregulated DEmiRNAs did not give a significantly different GSVA score between patients and controls (Figure 5D, Table 6), the score proved to differentiate the two groups well (Figure 5E). This suggests its potential as a diagnostic biomarker. Most core dysregulated DEmRNAs were up-regulated in schizophrenia compared to controls (Figure 5F, Table 7). The GSVA score based on core dysregulated DEmRNAs were significantly higher in patients (*p* = 0.0087, Figure 5G), and it differentiated patients from controls with good AUCs in the test set (GSE38484, AUC = 0.659) and validation set (GSE16930, AUC = 0.778) (Figure 5H).

**Table 6:** Autophagy-related DElncRNAs, DEmiRNAs, and DEmRNAs in the ceRNA network potentially involved in schizophrenia.

**DElncRNAs**	**DEmiRNAs**	**Autophagy-related DEmRNAs**
ANAPC10	miR-618	ANAPC10
PML	miR-423-5p	ATG2A
MAP1LC3A	miR-4532	BID
TRIM11	miR-593-3p	CDKN1A
CDKN1A	miR-3943	CDKN1B
MTA1	miR-296-5p	HDAC4
RAB18	miR-4640-3p	ITGA3
BID	miR-4723-3p	PML
CDKN1B		RAB4A
AHI1		SMAD4
RAB4A		TGFBR2
HDAC4		
SMAD4		
TMED10		
TGFBR2		

ceRNA, competing endogenous RNA; DE, differentially expressed;

*lncRNA, long non-coding RNA; miRNA, microRNA; SZ, schizophrenia*.

**Table 7:** The GSVA score of autophagy-related core DElncRNAs for schizophrenia.

**Sample**	**The SGVA score for lncRNA**
GSM943244	0.16523176
GSM943245	0.14017525
GSM943246	−0.0312192
GSM943247	0.4448873
GSM943248	−0.2400205
GSM943257	−0.0057562
GSM943258	−0.0528709
GSM943264	−0.2654075
GSM943265	0.37283798
GSM943266	−0.1413786
GSM943267	0.25294081
GSM943268	0.17203633
GSM943269	0.27556224
GSM943270	−0.1821881
GSM943271	0.22107073
GSM943272	−0.0750644
GSM943273	0.060905
GSM943274	0.45723052
GSM943275	0.4720365
GSM943276	−0.2633945
GSM943278	−0.3096199
GSM943279	0.50814003
GSM943280	0.3837298
GSM943281	0.04872864
GSM943304	−0.068256
GSM943305	−0.4797935
GSM943306	−0.3574512
GSM943307	0.0410844
GSM943308	0.29101183
GSM943309	0.17361072
GSM943310	−0.1717841
GSM943311	−0.183545
GSM943312	0.36433832
GSM943313	0.34957837
GSM943314	−0.1047499
GSM943315	−0.1102694
GSM943317	0.04221331
GSM943323	−0.2816547
GSM943324	0.4577875
GSM943325	0.15768867
GSM943326	−0.0543676
GSM943327	−0.2419679
GSM943333	−0.1737285
GSM943334	0.25010002
GSM943335	0.35667383
GSM943344	−0.1671558
GSM943345	0.33649746
GSM943346	−0.1248759
GSM943351	−0.3469978
GSM943353	0.22414036
GSM943354	0.11285974
GSM943355	0.36488225
GSM943356	0.11361519
GSM943357	0.04053765
GSM943358	0.09604639
GSM943359	−0.4523051
GSM943360	0.26266788
GSM943361	−0.2851021
GSM943362	−0.5041086
GSM943363	0.20250477
GSM943364	0.437185
GSM943365	0.29858339
GSM943366	0.35282093
GSM943367	−0.1602093
GSM943368	0.15444235
GSM943369	0.30437466
GSM943370	0.49672922
GSM943374	0.01369045
GSM943375	0.54751788
GSM943376	0.32566493
GSM943377	0.25941656
GSM943378	0.42973364
GSM943379	0.39773378
GSM943380	0.52339567
GSM943381	0.16793854
GSM943386	0.24790484
GSM943387	0.33336173
GSM943401	−0.5383407
GSM943402	−0.1192688
GSM943413	0.3574564
GSM943416	0.38225889
GSM943417	0.43491138
GSM943418	0.4146559
GSM943419	0.21660044
GSM943420	0.18849231
GSM943421	0.47769179
GSM943422	−0.2108428
GSM943423	0.24736208
GSM943424	−0.2980411
GSM943425	−0.3513198
GSM943426	0.13432676
GSM943427	−0.4502334
GSM943428	−0.1585483
GSM943429	−0.3833341
GSM943433	−0.0423108
GSM943434	−0.2169906
GSM943435	−0.4026923
GSM943436	−0.1357429
GSM943437	−0.3394727
GSM943438	−0.1165543
GSM943439	−0.03674
GSM943440	−0.2315041
GSM943441	−0.1892905
GSM943442	0.20327415
GSM943443	−0.0475085
GSM943444	−0.192442
GSM943243	−0.2628075
GSM943249	−0.3042399
GSM943250	−0.155386
GSM943251	0.27872752
GSM943252	−0.2753093
GSM943253	−0.0189304
GSM943254	−0.0317043
GSM943255	0.50690189
GSM943256	−0.325036
GSM943259	0.03048985
GSM943260	−0.0107852
GSM943261	−0.2204927
GSM943262	0.07026454
GSM943263	−0.5066773
GSM943277	−0.3784875
GSM943282	−0.1521591
GSM943283	−0.304476
GSM943284	0.19834163
GSM943285	−0.1352569
GSM943286	0.02463716
GSM943287	−0.1402883
GSM943288	−0.2367544
GSM943289	0.09349615
GSM943290	−0.0018526
GSM943291	−0.2289556
GSM943292	−0.2817136
GSM943293	−0.4355461
GSM943294	−0.3161009
GSM943295	0.02112862
GSM943296	−0.1410258
GSM943297	−0.4714194
GSM943298	−0.1162695
GSM943299	−0.3715334
GSM943300	−0.1911983
GSM943301	0.03913551
GSM943302	−0.2678182
GSM943303	−0.222806
GSM943316	0.43880862
GSM943318	−0.286218
GSM943319	−0.0885553
GSM943320	0.19620382
GSM943321	−0.217659
GSM943322	0.22623391
GSM943328	−0.1734389
GSM943329	−0.0224062
GSM943330	0.00323802
GSM943331	0.52188963
GSM943332	0.08645874
GSM943336	−0.3569525
GSM943337	−0.2940262
GSM943338	−0.4274155
GSM943339	−0.3371467
GSM943340	−0.6698214
GSM943341	0.24754192
GSM943342	−0.2931242
GSM943343	−0.3216991
GSM943347	−0.2958243
GSM943348	−0.0570591
GSM943349	−0.1881957
GSM943350	0.14668631
GSM943352	0.54968764
GSM943371	−0.0502532
GSM943372	0.26218968
GSM943373	0.14955676
GSM943382	−0.0134775
GSM943383	0.3173091
GSM943384	0.14585417
GSM943385	0.12580093
GSM943388	−0.1641372
GSM943389	0.08380789
GSM943390	−0.1966473
GSM943391	−0.1311395
GSM943392	−0.3472714
GSM943393	−0.4261266
GSM943394	−0.1352578
GSM943395	0.40150813
GSM943396	0.33829136
GSM943397	0.15849565
GSM943398	−0.3485866
GSM943399	0.09255036
GSM943400	−0.2833883
GSM943403	0.13860444
GSM943404	0.35069625
GSM943405	0.31839886
GSM943406	0.26587442
GSM943407	0.18053564
GSM943408	0.37962738
GSM943409	0.36431587
GSM943410	−0.2140642
GSM943411	0.03500473
GSM943412	−0.0196083
GSM943414	0.38688973
GSM943415	0.36851887
GSM943430	−0.2838209
GSM943431	−0.1250319
GSM943432	−0.3397713

*GSVA, gene set variation analysis; DElncRNA, differentially expressed lncRNA; lncRNA, long non-coding RNA*.

**Table 8:** The GSVA score of autophagy-related core DEmiRNAs for schizophrenia.

**Sample**	**The SGVA score for miRNA**
GSM1319273	−0.2902226
GSM1319274	−0.1687515
GSM1319275	0.36035455
GSM1319276	0.3414514
GSM1319277	0.39851853
GSM1319278	−0.3203811
GSM1319279	0.11212015
GSM1319280	0.51807188
GSM1319281	−0.139958
GSM1319282	0.43995331
GSM1319283	0.33134043
GSM1319284	0.46642465
GSM1319285	0.57104229
GSM1319286	0.32094183
GSM1319287	0.54250142
GSM1319258	−0.0306217
GSM1319259	−0.5113071
GSM1319260	−0.1971761
GSM1319261	−0.3622741
GSM1319262	0.16720548
GSM1319263	−0.3944622
GSM1319264	−0.6112523
GSM1319265	0.12238628
GSM1319266	0.12820827
GSM1319267	−0.4186762
GSM1319268	−0.5027713
GSM1319269	−0.6330539
GSM1319270	0.16355505
GSM1319271	−0.7776802
GSM1319272	−0.6659011

*GSVA, gene set variation analysis; DEmiRNA, differentially expressed miRNA; MiRNA, microRNA*.

**Table 9:** The GSVA score of autophagy-related core DEmRNAs for schizophrenia.

**Sample**	**The SGVA score for mRNA**
GSM943244	0.05578029
GSM943245	0.08487851
GSM943246	−0.0728044
GSM943247	0.4013904
GSM943248	−0.2001586
GSM943257	0.06661096
GSM943258	−0.3222998
GSM943264	−0.2131252
GSM943265	0.31110152
GSM943266	−0.142986
GSM943267	0.27328669
GSM943268	0.06219096
GSM943269	0.24486591
GSM943270	0.15258807
GSM943271	0.27207768
GSM943272	−0.1023498
GSM943273	−0.1609631
GSM943274	0.23711898
GSM943275	0.46183445
GSM943276	−0.287573
GSM943278	−0.0060698
GSM943279	0.51535806
GSM943280	0.37219589
GSM943281	0.06680157
GSM943304	0.00089912
GSM943305	−0.2960054
GSM943306	−0.2513268
GSM943307	0.10305816
GSM943308	0.25858371
GSM943309	0.20567306
GSM943310	−0.2788849
GSM943311	−0.3446586
GSM943312	0.52587649
GSM943313	0.15807803
GSM943314	0.12837864
GSM943315	0.09866567
GSM943317	−0.0838278
GSM943323	−0.3644114
GSM943324	0.40263811
GSM943325	0.02942296
GSM943326	−0.2819125
GSM943327	−0.4048892
GSM943333	−0.1865595
GSM943334	0.17542479
GSM943335	0.3231569
GSM943344	−0.2058041
GSM943345	0.60989966
GSM943346	0.11479437
GSM943351	0.06093777
GSM943353	0.18638731
GSM943354	0.29411436
GSM943355	0.235235
GSM943356	−0.0242047
GSM943357	0.23975762
GSM943358	−0.1424363
GSM943359	−0.4066069
GSM943360	0.39393186
GSM943361	−0.2461425
GSM943362	−0.3547428
GSM943363	−0.1365479
GSM943364	0.44915569
GSM943365	0.11189307
GSM943366	0.42270207
GSM943367	−0.3444578
GSM943368	0.06463209
GSM943369	0.00856351
GSM943370	0.55675699
GSM943374	−0.1613266
GSM943375	0.53619048
GSM943376	0.41747034
GSM943377	0.03905625
GSM943378	0.4434228
GSM943379	0.41551602
GSM943380	0.53482564
GSM943381	0.1764368
GSM943386	0.15127074
GSM943387	0.35253242
GSM943401	−0.3468688
GSM943402	−0.1340903
GSM943413	0.13059431
GSM943416	0.30840277
GSM943417	0.33667213
GSM943418	0.43882947
GSM943419	0.250528
GSM943420	0.13847615
GSM943421	0.32204832
GSM943422	−0.0172799
GSM943423	0.4513333
GSM943424	−0.289351
GSM943425	−0.2891064
GSM943426	0.46816806
GSM943427	−0.5037768
GSM943428	−0.0455529
GSM943429	−0.3205724
GSM943433	0.01648582
GSM943434	−0.3288121
GSM943435	−0.3348525
GSM943436	0.16527074
GSM943437	−0.1510088
GSM943438	−0.3746245
GSM943439	−0.0234759
GSM943440	−0.0446987
GSM943441	0.08441187
GSM943442	0.49931603
GSM943443	0.03503982
GSM943444	0.14282981
GSM943243	−0.2870646
GSM943249	−0.4050875
GSM943250	−0.1214628
GSM943251	−0.0474812
GSM943252	−0.0286448
GSM943253	0.10298879
GSM943254	−0.0106536
GSM943255	0.5477157
GSM943256	−0.3687687
GSM943259	−0.0827893
GSM943260	0.166678
GSM943261	−0.4602952
GSM943262	0.22238901
GSM943263	−0.3615747
GSM943277	−0.3873576
GSM943282	−0.0900237
GSM943283	−0.3750237
GSM943284	−0.0877004
GSM943285	−0.1695492
GSM943286	0.08766006
GSM943287	−0.3179648
GSM943288	−0.444477
GSM943289	−0.0792808
GSM943290	−0.059938
GSM943291	−0.3215349
GSM943292	−0.4619875
GSM943293	−0.4927457
GSM943294	−0.3328019
GSM943295	0.26444373
GSM943296	−0.3003772
GSM943297	−0.4231188
GSM943298	−0.1198662
GSM943299	−0.4330391
GSM943300	−0.3357985
GSM943301	−0.0537179
GSM943302	−0.3928497
GSM943303	−0.3438028
GSM943316	0.31658638
GSM943318	−0.4643914
GSM943319	−0.4886199
GSM943320	0.01997146
GSM943321	−0.35127
GSM943322	−0.05806
GSM943328	−0.2392504
GSM943329	0.08475588
GSM943330	−0.1014165
GSM943331	0.37657277
GSM943332	−0.0514438
GSM943336	−0.2891391
GSM943337	−0.3903604
GSM943338	−0.483639
GSM943339	−0.1835822
GSM943340	−0.5651424
GSM943341	0.28380396
GSM943342	−0.2616049
GSM943343	−0.453991
GSM943347	−0.1345205
GSM943348	−0.00573
GSM943349	0.14026447
GSM943350	0.39811744
GSM943352	0.67643425
GSM943371	0.02765994
GSM943372	−0.1065947
GSM943373	0.31023943
GSM943382	0.08623327
GSM943383	0.32344492
GSM943384	−0.015262
GSM943385	0.29628745
GSM943388	−0.2707811
GSM943389	−0.1824294
GSM943390	−0.2125335
GSM943391	−0.1242119
GSM943392	−0.3969811
GSM943393	−0.5011003
GSM943394	−0.1498812
GSM943395	0.36429884
GSM943396	0.2192296
GSM943397	0.00771798
GSM943398	−0.4154234
GSM943399	0.03564853
GSM943400	−0.3105713
GSM943403	0.20168907
GSM943404	0.29691025
GSM943405	0.26067473
GSM943406	0.41976051
GSM943407	0.27495679
GSM943408	0.31873066
GSM943409	0.32359379
GSM943410	−0.2609503
GSM943411	0.14871453
GSM943412	−0.0365365
GSM943414	0.28192595
GSM943415	0.07124618
GSM943430	−0.1285523
GSM943431	0.08576328
GSM943432	−0.1340666

*GSVA, gene set variation; DEmRNA, differentially expressed mRNA*.

## Discussion

Schizophrenia is a persistent mental illness that disrupts normal thinking, function and mobility, and it can seriously impact patients and their families. Current anti-schizophrenia drugs can treat only the symptoms of the disease (35). To deepen the understanding of pathology of SZ to guide the diagnosis and treatment, the present study exlpored a ceRNA network that may be related to the disease by altering the regulation of genes involved in autophagy.

At the core of the ceRNA network, we identified 15 lncRNAs, 8 miRNAs, 11 mRNAs, and 10 KEGG pathways. Several of these RNAs have already been associated with schizophrenia. The miRNA137, which maps to chromosome 1p21.3, appears to confer susceptibility to the disease (36), while miR-219 is significantly up-regulated in the dorsolateral pre-frontal cortex of patients (37). The lncRNA MIAT (38), also called Gomafu (39), is down-regulated in schizophrenia, and this lower expression appears to reduce the activity of neurons (40).

Among the 10 core KEGG pathways in our ceRNA network, nine have already been associated with schizophrenia: cell cycle (41), spinocerebellar ataxia (42), apoptosis (43), ErbB signaling (44), focal adhesion (45), endocytosis (46), adhesions junction (47), Wnt signaling (48), and MAPK signaling (49). Mammalian mTOR target mTOR complex 1 (mTORC1) phosphorylates Unc51-like autophagy-activated kinase to block the initiation of autophagy. Both AMPK and oxidative stress can activate the transcription factors EB, FOXO1/3, transcription factor 4, and NF-κB to turn on expression of the autophagy-activated kinase (50).

The results of this study show that based on the exploration of the ceRNA network in schizophrenia, eight core disorders of DEmiRNA (hsa-miR-423-5p, hsa-miR-4532, hsa-miR-593-3P, hsa-miR-618, hsa-miR-4723-3p, hsa-miR-4640-3p, hsa-miR-296-5p, and hsa-miR-3943) may play a role in the diagnosis and treatment of schizophrenia. This article provides some basis for the study of ceRNA in schizophrenia. A previous study showed that hsa-miR-423-5p expressed in brain and were associated with amyotrophic (50). Hsa-miR-296-5p can be used as the prognostic marker for anaplastic glioma, secondary and anterior glioma patient (51). These studies indicated that the miRNA may be used as biomarker for neurological diseases.

In short, this study provides deeper insights into the construction of lncRNA-miRNA-mRNA network involving autophagy-related genes in SZ, and provides new targets for the diagnosis of SZ patients. However, there are some limitations at present. Firstly, due to the small sample size of the lncRNA and mRNA verification sets, and the lack of miRNA verification sets. The expression profiles of lncRNA and mRNA are obtained from the same sample, but miRNA is obtained from a separate data set. The combination of two data sets into a network may lead to selection bias due to batch effect. Secondly, the results of our study only indicate that these ceRNA network may exist in patients with SZ. However, it needs further evidence whether ceRNA exists in SZ patients, with the help of systematic biological experiment *in vivo* or *in vitro*. Relevant molecular biology experiments are required to obtain more credible results.

## Conclusion

Our results suggest that the ceRNA network is involved in schizophrenia, which may deepen our understanding of the disease and guide the development of new treatments. The GSVA score based on the following eight core dysregulated DEmiRNAs may improve diagnosis of the disease: hsa-miR-423-5p, hsa-miR-4532, hsa-miR-593-3P, hsa-miR-618, hsa-miR-4723-3p, hsa-miR-4640-3p, hsa-miR-296-5p, and hsa-miR-3943.

## Data Availability Statement

The original contributions presented in the study are included in the article/Supplementary Material, further inquiries can be directed to the corresponding authors.

## Author Contributions

RL, QW, YQ, DZ, and CL designed the study and contributed to drafting the manuscript. RL, QW, YQ, YM, LW, HW, RM, DZ, and CL collated data and carried out data analyses. All authors have read and approved the final submitted manuscript.

## Conflict of Interest

The authors declare that the research was conducted in the absence of any commercial or financial relationships that could be construed as a potential conflict of interest.
