# Supplementary figures and images for: A Potential Autophagy-Related Competing Endogenous RNA Network and Corresponding Diagnostic Efficacy in Schizophrenia
Source: Front Psychiatry. 2021 Feb 23;12:628361. doi: 10.3389/fpsyt.2021.628361 (PMC7940829; doi:10.3389/fpsyt.2021.628361)

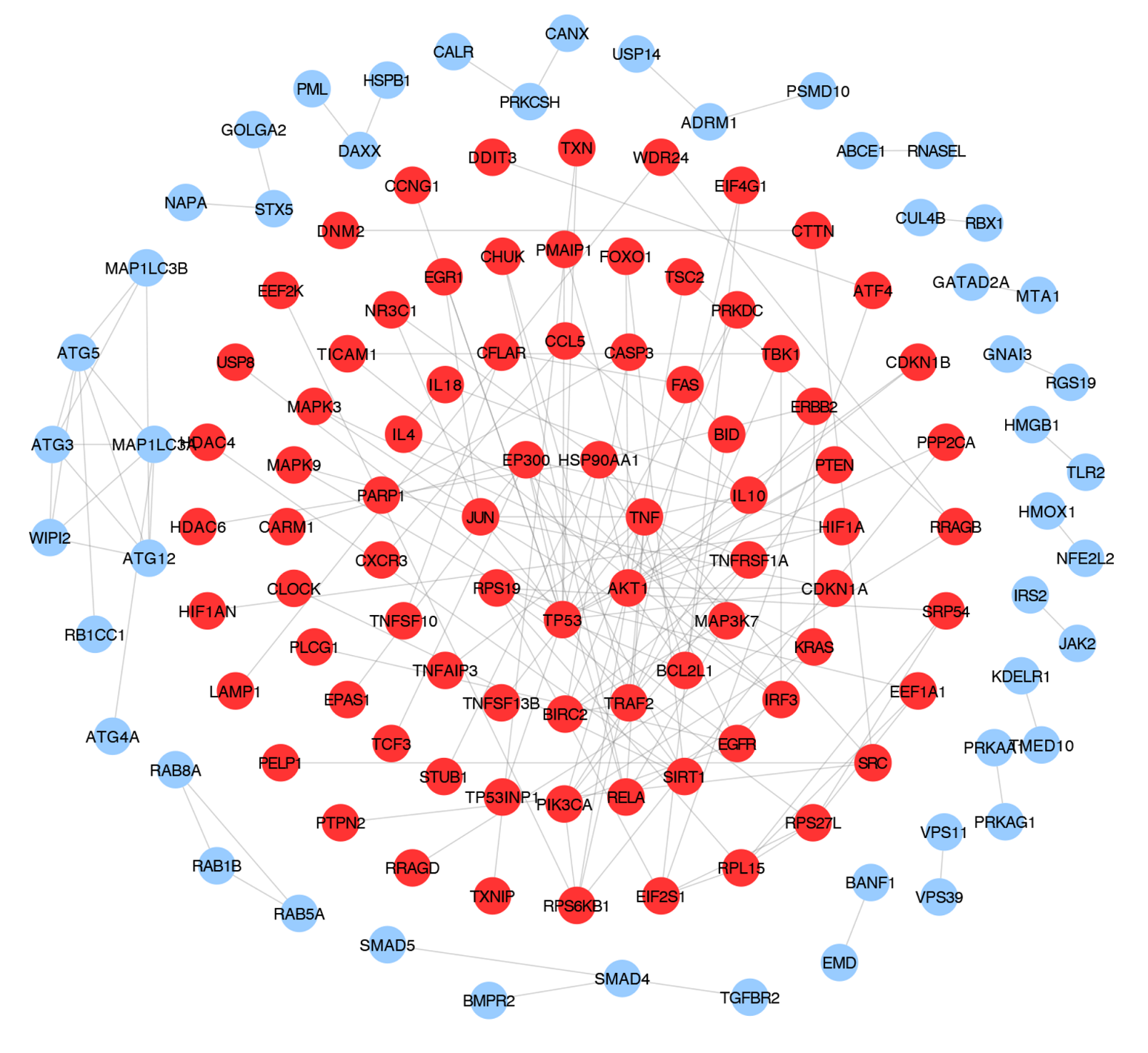

Supplement: Supplementary file 1 [file Image_1.tif]
